# Ultra-High-Velocity Penetration Performance of Lightweight W-Based Ceramic Alloy Rod Penetrator Against Concrete Targets

**DOI:** 10.3390/ma19132924

**Published:** 2026-07-07

**Authors:** Rui Yang, Yun Zhu, Jianping Fu, Kai Ren, Yupeng Guo, Shuai Liu, Yuyu Ma

**Affiliations:** 1 School of Mechanical and Electrical Engineering, North University of China, Taiyuan 030051, China; 20230214@nuc.edu.cn (R.Y.);; 2 Shate Key Laboratory of Target Vulnerability Assessment, Defense Engineering Institute, AMS, PLA, Luoyang 471000, China; 3 School of Artificial Intelligence, Hebei University of Technology, Tianjin 300401, China; 4 State Key Laboratory of Intelligent Power Distribution Equipment and System, Hebei University of Technology, Tianjin 300401, China; mayuyu@hebut.edu.cn

**Keywords:** lightweight W-based alloy, ceramic microspheres, ultra-high-velocity penetration, rod penetrator, concrete target, penetration performance

## Abstract

**Highlights:**

**What are the main findings?**

- A new type of lightweight tungsten-based ceramic alloy was fabricated.
- This lightweight tungsten-based ceramic alloy achieves both weight reduction and enhanced penetration performance of armor-piercing penetrators.
- The improved theoretical penetration calculation model enhances the prediction accuracy of the ultra-high-velocity penetration behavior of penetrators.

**What are the implications of the main findings?**

- This work offers theoretical guidance for the design of lightweight high-performance penetrators.
- It optimizes the penetration theoretical model and improves engineering prediction reliability.
- This study lays a foundation for further research on ultra-high-velocity penetration.

**Abstract:**

Aiming at the reduction in penetration depth caused by the deformation and fracture of conventional 90W-Ni-Fe rod penetrators during ultra-high-velocity penetration, a lightweight tungsten-based ceramic alloy was fabricated in this study. Ballistics tests were conducted to verify the penetration performance of the novel lightweight alloy against concrete targets, and the existing theoretical penetration model was modified accordingly. The results indicate that, within the impact velocity range of 1400~1750 m/s, the penetration depth of both rod penetrators presents an initial increasing and subsequent decreasing trend, with an extreme value near 1625 m/s and a dimensionless penetration depth *X*_p_/*L* close to 4. Compared with the traditional 90W-Ni-Fe alloy, the lightweight tungsten-based ceramic alloy penetrator achieves a mass reduction of 5.7%. In the impact velocity range from 1408.0 m/s to 1743.5 m/s, its penetration depth increases by 6.22%~10.58%, and the improvement becomes more significant with the increase in impact velocity. For the modified theoretical model, the predicted ultimate penetration depth of the lightweight alloy rod penetrator increases by 8.85%, while the average mass loss rate and average erosion rate decrease by 12.09% and 17.65%, respectively. The error between theoretical calculations and experimental data is within 5%.

## 1. Introduction

As the requirement for penetration velocity continues to grow, tungsten alloy long-rod penetrators are prone to severe deformation, fragmentation and mass ablation. This gives rise to an adverse phenomenon in which the penetration depth stops increasing and even drops with rising impact velocity. To boost the hypervelocity penetration capacity, novel penetrator materials tailored for ultra-high-speed impact environments are urgently required. Of particular interest are self-sharpening tungsten-based composites characterized by high density, superior specific kinetic energy and pronounced adiabatic shear susceptibility.

Tungsten-based alloy rods are most widely used in the realm of ultra-high-speed (above 1400 m/s) penetrators [1,2,3,4]. Under ultra-high-speed conditions, the penetration mode of such materials is semi-fluid, and the dimensionless penetration depth has an extremum, resulting in lower penetration efficiency [5,6,7]. To enhance the penetrating power of tungsten alloy rods, various oxides are added [8,9], which not only reduces the grain size but also improves its mechanical properties and adiabatic shear performance [10,11,12]. The microstructure of the tungsten alloy is optimized by adjusting the alloy composition, sintering process, and deformation strengthening post treatment to achieve better overall mechanical properties, enabling the formation of adiabatic shear bands during penetration and enhancing its penetration depth and other performance indicators. However, the final properties of the tungsten alloy vary with the composition, content, and fabrication process of the alloy [13]. Alloy strengthening is a primary method to reduce the brittleness of tungsten-based ceramic alloys. Recent studies worldwide have shown that [14,15,16] refined grain size can effectively enhance the mechanical properties of high-density tungsten alloys. By adding grain growth inhibitors to adjust the grain size, the penetration performance of high-density tungsten alloys can be improved. For example, adding rare-earth elements such as La, Ce, or Y and oxides like ThO_2_, Y_2_O_3_ or A1_2_O_3_ can inhibit grain growth and reduce interfacial segregation, thus improving the structure and mechanical properties of tungsten alloys [17]. This material, while ensuring the dynamic compressive strength and plasticity of tungsten-based ceramic alloys, reduces their tensile strength to achieve a secondary damage effect after penetration. The use of La_2_O_3_/ZrC composite ceramic powder to enhance tungsten results in a 30% increase in strength over materials strengthened by a single phase [18].

Plenty of investigations on tungsten-based composite penetrators have been conducted in recent years. Singh et al. summarized the microstructure, self-sharpening behavior and ballistic performance of conventional tungsten heavy alloys, and pointed out the obvious drawbacks of monolithic W-Ni-Fe alloys including large plastic deformation and serious mass ablation under ultra-high-speed impact [19]. Wu et al. built a quantitative coupling relationship between the impact velocity and quasi-steady penetration depth of tungsten long-rod penetrators, which provides theoretical support for depicting penetration dynamic evolution [20]. Patel et al. systematically reviewed the manufacturing approaches, microstructure control and mechanical performance of ceramic particle modified tungsten matrix composites [21]. Cao et al. investigated sintering procedures, interfacial phase constitution and microstructure-performance correlations of high-tungsten Al_2_O_3_/W composites [22]. Zhu et al. adopted XRD tests to illustrate the grain refinement and strength–ductility synergy mechanism triggered by dispersed oxide second phases [23]. Wartacz et al. characterized the interfacial adhesion and high-temperature anti-ablation capacity of fiber-reinforced tungsten composites, offering references for EDS elemental mapping analysis in this work [24]. Liu et al. clarified the erosion and fracture evolution of tungsten alloy projectiles penetrating concrete targets, which helps to evaluate its civil application value in emergency obstacle removal [25]. Sheng et al. proposed a self-sharpening penetration model for tungsten high-entropy alloys to enrich the semi-fluid penetration theoretical framework [26]. Wang et al. revealed the strengthening effect of TiC-ZrC dual ceramic particles on tungsten substrate and quantified their promotion on hardness and impact resistance [27]. Cui et al. implemented multiple repeated ballistics tests for tungsten penetrators and analyzed data dispersion via standard deviation of parallel samples [28]. Krolczyk et al. expanded the application scope of tungsten composites to wear-resistant parts for civil geological drilling engineering [29]. Ni et al. developed a low-temperature rapid sintering route for ultrafine refractory tungsten materials, supplying innovative guidance for sintering parameter optimization [30]. Compared with various tungsten-based materials reported in the above literature, the lightweight 2.5 wt.% W–Al_2_O_3_ composite fabricated by hydrogen atmosphere liquid-phase sintering in the present study delivers a larger dimensionless penetration depth, lower mass loss rate and milder surface erosion under identical ultra-high-velocity impact, successfully addressing the technical bottlenecks of severe lateral deformation and excessive ablation loss that widely exist in traditional tungsten alloys and low-filler ceramic reinforced tungsten composites.

Although numerous investigations have been carried out on reinforced tungsten-based composites [31,32,33,34], their application as ultra-high-velocity penetrators, especially against concrete targets, has rarely been reported. Restricted by the capacity of launch facilities, large-scale ultra-high-speed penetration experiments are hard to implement, resulting in a scarcity of reliable experimental data. Relevant research concerning long-rod penetrators impacting concrete at ultra-high velocities is still in the preliminary stage, with insufficient theoretical frameworks and experimental verifications. In this work, sintered alumina ceramic particles are incorporated into tungsten alloys to synergistically take advantage of the respective merits of ceramic and tungsten matrix under hypervelocity impact. The novel tungsten-based ceramic composite is designed for long-rod projectiles. By tuning material brittleness and changing the propagation route of internal cracks, the material can facilitate the development of the semi-fluid penetration stage when striking concrete targets.

## 2. Mechanical Properties of Tungsten-Based Ceramic Composites

To improve the hypervelocity penetration performance of conventional tungsten alloys, a novel tungsten-based ceramic composite is fabricated by liquid-phase sintering. Mechanical testing, metallographic characterization and fracture morphology analysis are subsequently performed on the alloy.

### 2.1. Microstructure

**Figure 1:**
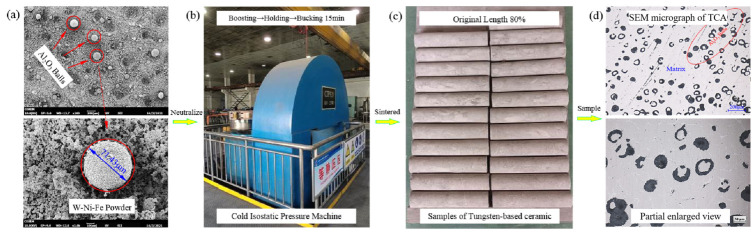
The preparation process of the new tungsten-based ceramic alloy material and microstructure. (**a**) SEM image showing the distribution of ceramics in tungsten alloy powder after mixing; (**b**) Cold isostatic pressing process; (**c**) Green billet of sintered tungsten-based ceramic alloy; (**d**) SEM image of ceramic distribution in the sintered material.

Material preparation was performed firstly. Sintered alumina ceramics with a mass fraction of 2.5% were incorporated into 90W tungsten alloy powder, followed by sufficient homogenization. Steel balls were adopted as milling media to reduce impurity elements in raw materials. The fabrication procedure and corresponding microstructure of the novel tungsten-based ceramic alloy are illustrated in Figure 1 [35]. The lightweight tungsten-based ceramic alloy was sintered in a hydrogen atmosphere to avoid oxidation. The sintering process was carried out with a heating rate of 5 °C/min, a sintering temperature of 1480 °C, and a holding dwell time of 120 min. These optimized sintering parameters ensure sufficient liquid-phase wetting and interfacial bonding between the tungsten matrix and ceramic reinforcement, and guarantee the densification and uniform microstruc ture of the composite material.

The sintering process of tungsten-based ceramic alloys consists of three consecutive stages: liquid-phase particle rearrangement, solid-phase precipitation, and skeleton formation [36]. During heating, nickel and iron powders undergo mutual diffusion at a relatively low temperature to form a binary solid solution, while solid-phase diffusion simultaneously occurs at the interfaces between tungsten particles and the binder-phase powders. When the temperature reaches the Ni–Fe binary eutectic point, a liquid phase forms within the powder system. Tungsten particles are nearly suspended in the generated liquid phase and migrate under the driving force of liquid surface tension. Meanwhile, capillary force arising from the liquid phase within particle pores, together with liquid viscous flow, promotes the positional adjustment and rearrangement of tungsten particles, ultimately achieving a dense particle packing state. Owing to the high solid solubility of tungsten in nickel, iron, and their alloys, tungsten atoms on particle surfaces continuously dissolve into the liquid phase during liquid-phase sintering. In parallel, supersaturated tungsten atoms in the liquid phase precipitate and redeposit on the surfaces of large tungsten particles. This dynamic dissolution–precipitation mass transfer process persists continuously, with the liquid phase acting as the major mass-transport medium. Upon the completion of solid dissolution and precipitation, spherical tungsten particles are uniformly dispersed within the binder phase, and the sintering system enters the solid skeleton formation stage. At this stage, adjacent tungsten particles contact and undergo solid-phase sintering, forming stable interparticle bonding. Grain growth and skeleton densification are further realized via atomic diffusion. During the entire sintering process, alumina ceramic particles are embedded within the solid–liquid mixed matrix and ultimately immobilized in the final sintered microstructure.

All fabricated alloy specimens were mounted and encapsulated following standard metallographic procedures. A complete surface pretreatment workflow, including rough grinding, fine grinding, and mirror polishing, was performed in sequence. The flat and smooth surfaces of polished specimens were then directionally etched using a self-configured corrosive reagent. After etching, cleaning, and natural air-drying, an optical metallurgical microscope was employed to characterize the microstructural morphology and phase distribution of the alloy specimens prepared under different processing conditions [37]. The etching solution was formulated with deionized water, 10% hydrogen peroxide, and ammonia water at a volume ratio of 2:2:1, with the etching time strictly controlled between 30 s and 40 s. Metallographic observations reveal that the ceramic particles undergo no obvious deformation during liquid-phase sintering, maintaining a stable suspended distribution state within the alloy matrix.

**Figure 2:**
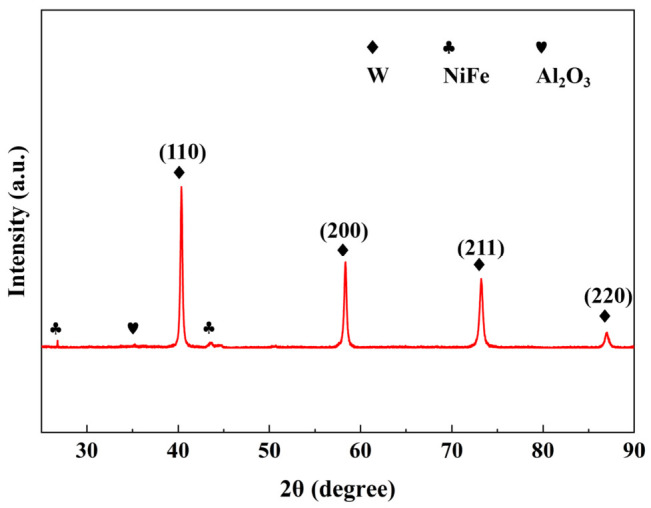
XRD patterns of tungsten-based ceramic alloy.

XRD phase characterization was carried out on the tungsten-based ceramic alloy with a 2.5 wt.% ceramic phase, as shown in Figure 2. The XRD pattern is dominated by characteristic diffraction peaks of metallic tungsten, which correspond to the typical crystal planes (110), (200), (211) and (220) of tungsten matrix. The diffraction peaks feature sharp shapes and favorable symmetry, revealing the excellent crystallinity of the matrix. Affected by the low doping amount of ceramic phase and the fluorescence scattering effect of tungsten matrix, a flat baseline platform appears in the range of 2θ < 30°, where weak characteristic diffraction peaks of alumina can be observed. No tungsten–aluminum intermetallic compounds or miscellaneous oxide peaks are detected in the whole scanning range. It is verified that the composite is only composed of a tungsten matrix and ceramic reinforcing phase with pure phases and no harmful reaction products.

**Figure 3:**
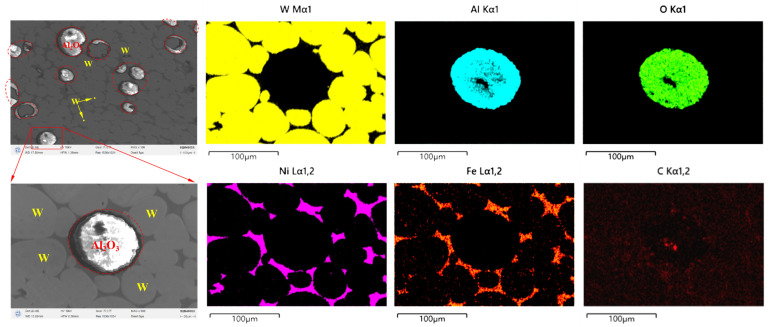
EDS spectra of tungsten-based ceramic alloy.

EDS spectrum tests were adopted to analyze the elemental composition and distribution characteristics of the lightweight tungsten-based ceramic alloy, as shown in Figure 3. EDS results illustrate that the composite mainly contains five characteristic elements including W, Al, O, Ni and Fe without any impurity elements, which is fully consistent with the designed material formula. W, Ni and Fe are the core elements of 90W alloy matrix, distributing continuously and uniformly to form the main skeleton of the material. Al and O elements are enriched in the areas of ceramic particles, which correspond to alumina ceramic reinforcing phases and prove the successful compounding of the ceramic phase into the tungsten alloy matrix. Elemental mapping results demonstrate that ceramic particles are uniformly dispersed without agglomeration or segregation. Elements of the matrix and reinforcing phase interpenetrate mutually with excellent interfacial bonding. The above results effectively confirm the reliability of the preparation process in this work and provide a microstructural basis for the improvement of mechanical and penetration properties of the composite.

### 2.2. Mechanical Properties

The tensile strength of the specimens was measured by a mechanical tensile testing machine. The tensile loading rate was set to 1 mm/min, and the tensile strain rate was 1.1 × 10^−3^ s^−1^. The impact properties of the material were tested using a manual pendulum impact testing machine, utilizing notch-free standard specimens measuring 10 mm × 10 mm × 55 mm. Performance tests were conducted on the tungsten-based ceramic alloy samples used for rod projectiles. Parallel specimens from the same batch were used in all tests. Elongation and reduction in the area were measured synchronously during the tensile tests. Impact toughness was tested on standard specimens in accordance with the GB/T 229-2020 Metallic materials—Charpy pendulum impact test [38]. Five parallel specimens were prepared for each material, and the original test data are listed in Table 1.

From the data presented in the table, it can be observed that the addition of alumina ceramics leads to a decrease in tensile strength, elongation, and impact toughness. The inclusion of ceramics reduces the mechanical properties and increases brittleness, making the material more characteristic of ceramics. The tungsten-based ceramic alloy consists of a multiphase alloy comprising a 90W alloy matrix and alumina ceramic particles. The physical properties of this alloy also adhere to the rule of summation between different phases in composite materials [39].

**Figure 4:**
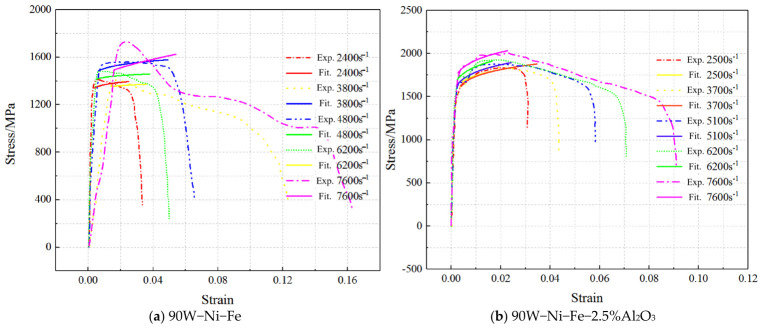
Stress–strain fitting curves for four materials at different strain rates.

Dynamic loading tests of the two materials were conducted using the split Hopkinson pressure bar (SHPB) system to investigate their strain rate effects under high-speed dynamic loading. The parameter *C* in the Johnson–Cook constitutive equation was defined as the strain rate sensitivity coefficient. The experimental curves at various strain rates were fitted, and the fitted stress–strain curves of the two materials are presented in Figure 4. The average of the fitted values was taken as the final *C* value. The compressive *C* values at different strain rates are listed in Table 2, with the reference strain rate set to 10^−3^ s^−1^. By calculating the arithmetic mean of the data in the table, the strain rate sensitivity coefficients of conventional 90W alloy and tungsten-based ceramic alloy were determined to be 0.0342 and 0.0462, respectively.

Dynamic tensile tests were carried out on conventional 90W alloy and tungsten-based ceramic alloy. The Johnson–Cook (J-C) constitutive parameters of the two materials were fitted based on the experimental data. Both materials exhibit obvious strain rate effects under high-rate compressive loading. Within the tested strain rate range of 2400 s^−1^ to 7600 s^−1^, the ultimate strength of conventional 90W alloy and tungsten-based ceramic alloy increases by 27.54% and 25.43%, respectively. Under high-velocity impact, the ceramic micro-particles inside the matrix hinder material deformation and induce localized shear deformation along slip lines, forming a self-sharpening effect. Compared with the conventional 90W alloy, the tungsten-based ceramic alloy shows a 13.64% increase in strength and a 43.75% decrease in ductility.

### 2.3. Fracture Analysis

**Figure 5:**
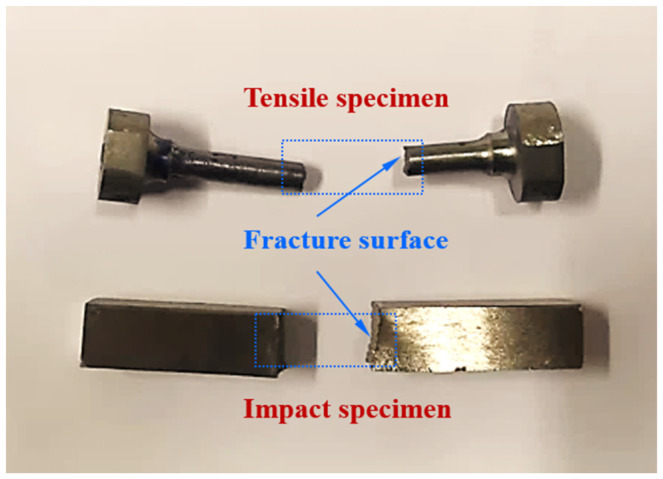
Macro fracture of standard specimen of tungsten-based ceramic alloy.

The typical macroscopic fracture morphology of the sintered tungsten-based ceramic alloy is presented in Figure 5. The fracture surface is relatively flat and bright, exhibiting prominent crystalline features and typical brittle fracture characteristics. The measured fracture reduction in the area of the alloy is 5%.

**Figure 6:**
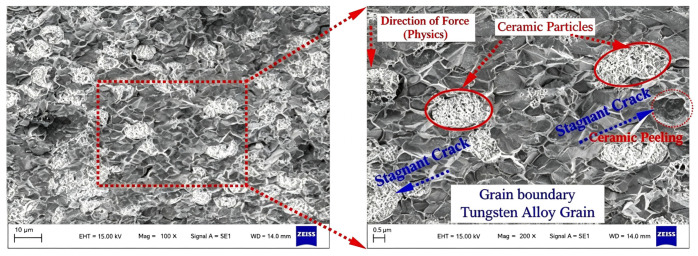
Distribution of Al_2_O_3_ ceramic particles in the fracture area.

Further microstructural characterization of the fracture region was performed via scanning electron microscopy (SEM). The distribution of alumina ceramic particles on the fracture surface is illustrated in Figure 6. It can be observed that alumina ceramic particles are uniformly dispersed within the tungsten alloy matrix, and a large number of shallow pits and microvoids are distributed across the fracture surface.

## 3. Ballistic Impact Experiment

### 3.1. Experimental Method

**Figure 7:**
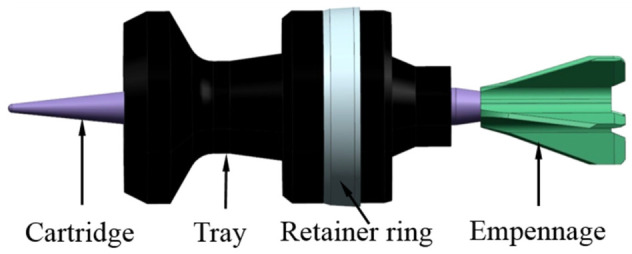
Structure of tungsten-based ceramic alloy rod-type bullet.

In this study, rod-shaped projectiles were fabricated separately using conventional tungsten alloy and novel tungsten-based ceramic alloy, and their penetration performance against C45 concrete targets was comparatively investigated. The projectile structure consists of a core body, a base, tail fins, and a fastening ring, among which the core body serves as the primary penetration component. The core body is designed with a diameter of 24.5 mm and a length of 321 mm. The structural configuration of the tungsten-based ceramic alloy rod projectile is presented in Figure 7.

**Figure 8:**
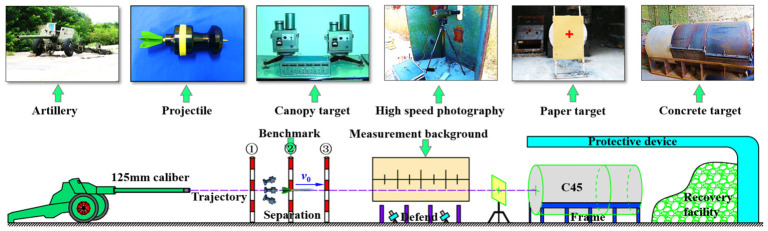
Intrusion power test site layout.

Penetration tests against C45 concrete targets were carried out via wobble tests with preset impact velocities and fixed penetration distances. The detailed test procedures are described as follows: C45 concrete targets with dimensions of Ø1.5 m × 1 m and aftereffect targets of identical size were hoisted and precisely aligned. The central axis of the target impact surface was strictly calibrated to be coaxial with the gun muzzle axis. The impact face of each target was vertically arranged and perpendicular to the horizontal reference plane, meeting the assembly accuracy requirements of test tooling. A wobble paper target was placed 5 m in front of the concrete target to monitor the flight attitude of projectiles during impact. The penetration depth (DOP) of conventional 90W tungsten alloy and novel tungsten-based ceramic alloy (TCW) rod projectiles was systematically tested and compared. The impact velocity ranged from 1400 m/s to 1750 m/s. The penetration behaviors of the two types of projectiles were analyzed under hypervelocity conditions, and the overall test setup is demonstrated in Figure 8.

### 3.2. Test Results

**Figure 9:**
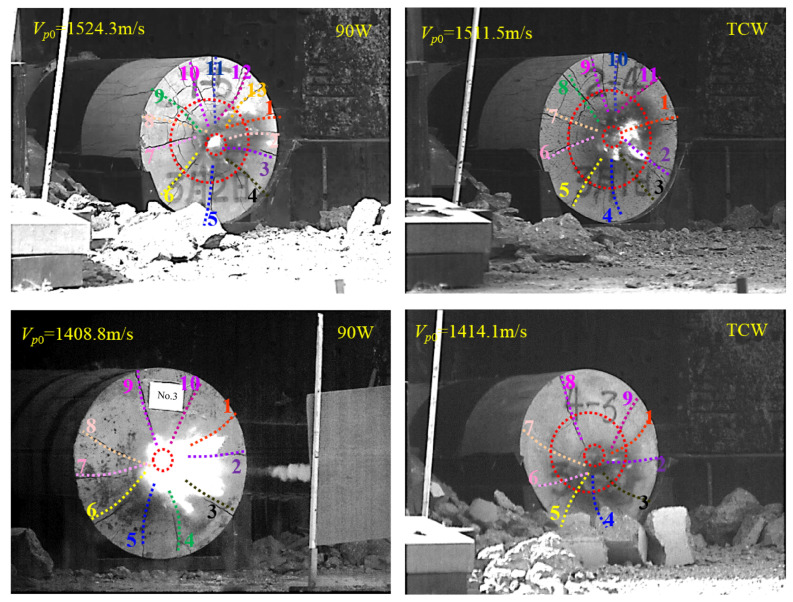
Crack expansion of test target body. Dashed lines represent radial cracks, dashed circles represent circumferential cracks, and numbers denote the quantity of cracks. TCW, tungsten-based ceramic alloy.

During hypervelocity penetration, the concrete material within the crushing zone around the penetration trajectory bears triaxial compressive stress, whereas the region far from the trajectory is dominated by tensile stress. Once the tensile stress exceeds the ultimate tensile strength of concrete, microcracks initiate and continuously propagate toward the target boundary. With the increase in impact velocity, both radial and circumferential primary cracks on the target surface grow in quantity. Sustained excitation of stress waves and energy input drives the further development and expansion of these cracks, which eventually intersect and fragment the concrete target into massive debris. A portion of the projectile’s impact energy is converted into the kinetic energy of target fragments, enabling the debris to disperse outward in all directions. Fragments near the ground are constrained by the target mounting frame and maintain relatively intact morphology, while debris in the other three quadrants is severely broken and splashes away from the target body. In addition, circumferential cracks are mainly distributed along the annular region at approximately half of the target radius. High-speed photography measurement shows that the propagation velocity of radial primary cracks reaches approximately 1000 m/s. Axial cracks propagate along the projectile penetration direction, which is attributed to the continuous stress wave excitation at the projectile–target interface. These axial cracks always propagate ahead of the projectile, inducing pre-damage in the undisturbed target material, and their propagation velocity is highly consistent with the projectile impact velocity. As the projectile velocity decays, the crack-affected zone at the projectile tip shrinks gradually, and the crack propagation velocity decreases with increasing distance from the penetration axis. The crack propagation morphology of the tested concrete target is presented in Figure 9.

The penetration test data of rod projectiles fabricated from the two materials against concrete targets are summarized in Table 3. The impact velocity error for each comparative test group is controlled within ±20 m/s to ensure test consistency. Specifically, specimens W–I to W–IV denote conventional tungsten alloy rod projectiles, while specimens T–I to T–IV correspond to tungsten-based ceramic alloy rod projectiles. Comparative analysis was conducted to evaluate the penetration performance of the two projectile types, and the penetration characteristics of tungsten-based ceramic projectiles were systematically explored within the impact velocity range of 1400 m/s to 1750 m/s.

Compared with the conventional 90W-Ni-Fe penetrator, the lightweight tungsten-based ceramic alloy penetrator achieves a mass reduction of 5.7%. The penetration tests reveal that both types of projectiles exhibit a consistent variation trend in penetration performance: the penetration depth increases first and then decreases with rising impact velocity. The penetration performance reaches an optimal value at approximately 1625 m/s, followed by a sharp decline, with the maximum dimensionless penetration depth (*X*_p_/*L*) approaching 4. Throughout the test velocity range, the tungsten-based ceramic alloy projectiles deliver superior penetration performance compared with traditional tungsten alloy projectiles, with a penetration depth improvement ranging from 6.22% to 10.58%. Such penetration enhancement becomes more prominent as the impact velocity increases, demonstrating that the proposed tungsten-based ceramic alloy is more applicable for ultra-high-velocity penetration against concrete targets.

Further analysis of the crater diameter and penetration depth of residual holes on target plates indicates that, for tungsten-based ceramic alloy projectiles, the crater diameter increases monotonically, while the penetration depth follows an initial increase and subsequent decrease with the elevation of impact velocity. The maximum penetration depth of ceramic-modified projectiles is 10.58% higher than that of conventional tungsten alloy counterparts. The inferior penetration performance of traditional 90W tungsten alloy projectiles is mainly attributed to nose deformation during penetration. Under a given initial kinetic energy, the penetration hole volume remains relatively stable, and the nose deformation of tungsten alloy projectiles induces excessive energy dissipation in radial crater expansion, which ultimately reduces the effective penetration depth.

### 3.3. Result Analysis

**Figure 10:**
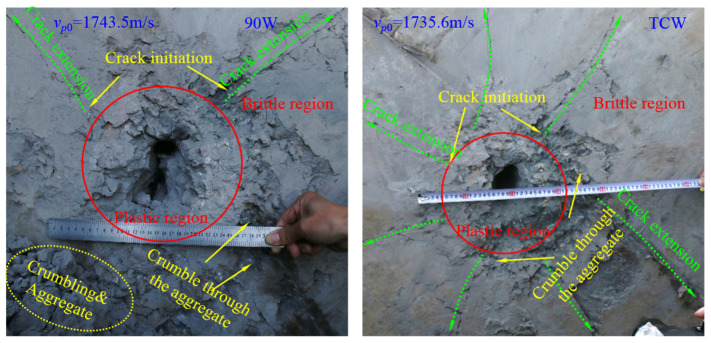
Local diagram of typical open pit.The green dotted arrows represent the crack propagation direction; the inner area inside each red circle stands for the plastic zone, while the outer area outside the circle represents the elastic zone. The yellow text labels mark spalling positions and crack initiation points.

During the penetration process, radial primary cracks initiate at the projectile–target contact point and propagate outward to the surrounding target region. With the continuous penetration of the projectile, secondary cracks generate around the primary radial cracks and rapidly extend toward the free boundaries of the target, accompanied by continuous crack widening. Subsequently, circumferential cracks emerge and interconnect with radial primary cracks, thoroughly fragmenting the target matrix into discrete debris. Constrained by the internal stress field, these fragmented particles are radially extruded and ejected outward, resulting in severe pulverization of the target material. The dominant edge cracks further penetrate through the target and extend to the rear surface, inducing central spalling on the target rear face along the penetration direction. The front region of the target is completely pulverized, producing an overall backward displacement of 260 mm. The local morphological characteristics of craters formed on typical target plates under ultra-high-velocity penetration are displayed in Figure 10. The target damage region consists of both brittle failure zones and plastic deformation zones, where the mortar and coarse aggregate of the concrete target peel off extensively, and cracks continuously initiate and propagate along the spalling interfaces.

**Figure 11:**
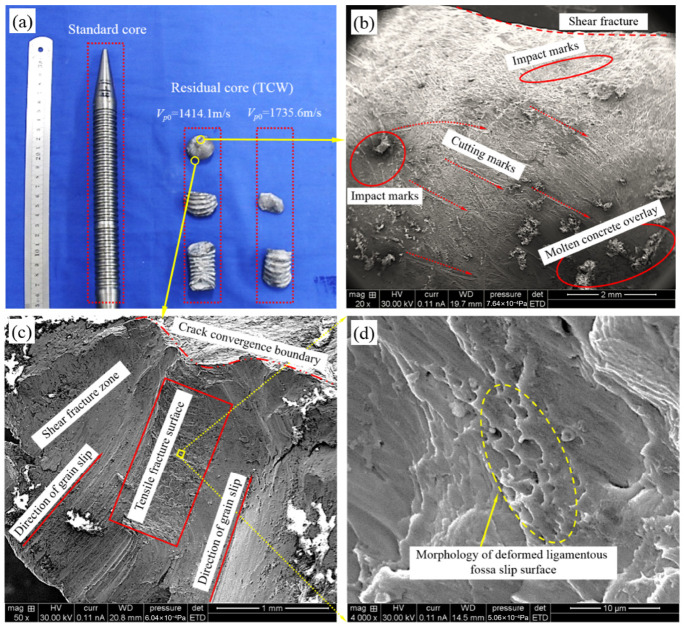
Residual cores recovered and bonded phase deformation tough nest. (**a**) Macro photographs of intact standard core and residual TCW fragments recovered under impact velocities; (**b**) Low-magnification SEM morphology of residual core surface, showing shear fracture zone, impact marks, cutting marks and molten concrete overlay; (**c**) Medium-magnification SEM image of fracture surface, presenting crack convergence boundary, shear fracture zone, tensile fracture surface and grain slip directions; (**d**) High-magnification SEM micrograph of tensile fracture area, displaying the morphology of deformed ligamentous fossa slip surface marked by yellow dashed ellipse.The green dotted arrows represent the crack propagation direction; the inner and outer regions of red circles correspond to the plastic zone and elastic zone, respectively. The yellow text labels mark spalling sites and crack initiation points.

Post-test inspection of recovered projectiles indicates that rod projectiles undergo segmental fracture during ultra-high-velocity penetration, and the degree of fragmentation intensifies with increasing impact velocity. The projectile tips present oval erosion morphologies, and fracture positions exhibit obvious material deformation and bending characteristics. The projectile bases are retained at the crater bottom, where the diameter of the loose damage zone is approximately three times the original projectile diameter. All recovered residual projectiles were ultrasonically cleaned with alcohol and subsequently characterized using a Quanta 400 scanning electron microscope. Quanta 400 scanning electron microscope (SEM) was manufactured by Thermo Fisher Scientific (FEI), Hillsboro, USA. Microscopic observations reveal that the residual penetration surfaces of projectile tips are uneven and covered with numerous depth-differentiated grooves. Most grooves present fine axial striped distribution along the projectile axis, while a small number of arc-shaped grooves are also observed, and a large quantity of target material adheres to the residual projectile surfaces. The microscopic morphology demonstrates intense projectile–target interaction during penetration, including cutting and extrusion damage induced by target particles as well as typical penetration damage features. Severe interfacial friction between the projectile and target causes thermal softening of the projectile’s surface layer and fusion bonding of target particles, ultimately leading to massive target material adhesion on the projectile surface. The distinct impact damage features further verify an extremely short projectile–target contact duration and an ultra-high deformation rate. The formed grooves possess complete and clear morphological characteristics with distinguishable bottom and sidewall features, and dense tiny pits are distributed around the fine striped grooves on residual projectile surfaces. The microscopic scanning results of the recovered projectiles are presented in Figure 11.

Microscopic observation of residual projectile fracture surfaces shows dominant disordered groove-like damage marks consisting of mixed striped and arc-shaped morphologies. Longitudinal cracks distributed at the fracture edges are mainly characterized by W-W particle pull-out, accompanied by a small number of tungsten particles with typical cleavage fracture features. In local regions near the outer penetration surface of fractured projectiles, the binder phases between tungsten particles present distinct deformed dimple morphologies. This unique fracture behavior originates from the intrinsic properties of tungsten-based ceramic alloys. The material possesses high density, hardness and structural strength, which endows it with predominant brittle fracture characteristics and a low deformation tendency during hypervelocity penetration. Fe, Ni and Co serve as the binder phases of tungsten-based ceramic alloys, among which γ-Fe, α-Co and Ni all exhibit face-centered cubic (FCC) crystal structures. FCC-structured materials contain abundant slip systems, and the complex phase transformation behavior of multi-element alloy binder phases, especially near the penetration surface, induces localized plastic deformation and deformed dimple morphologies, demonstrating excellent local plasticity and toughness. Furthermore, the incorporation of sintered alumina ceramic optimizes the strength–toughness compatibility of the alloy system, which further promotes the formation of dimple morphologies in the binder phase.

During hypervelocity impact, the plastic deformation work of projectiles is rapidly converted into heat energy. Due to the extremely short impact duration, the energy conversion process approximates an adiabatic condition, facilitating the generation of adiabatic shear bands. The thermal softening effect induced by adiabatic heating dominates over strain hardening and strain-rate hardening, thereby promoting the formation of adiabatic shear bands. The formation of shear bands is primarily governed by the thermal conversion efficiency of plastic work, which is closely related to the material’s microstructure, chemical composition and atomic arrangement. The prominent deformed dimple morphologies observed near the penetration surface of residual projectiles confirm significant thermal effects in these regions. Elevated temperatures effectively promote substantial plastic deformation of the surrounding binder phases during penetration.

## 4. Ultra-High-Velocity Penetration Theory of Rod Projectiles

### 4.1. Projectile Penetration Depth

The mainstream theoretical calculation models for penetration mechanics are mainly divided into two categories: cavity expansion theory and hydrodynamic penetration theory. Among them, the cavity expansion theory is mostly applied to solve the mechanical response of typical solid elastoplastic penetration, while the hydrodynamic penetration theory is suitable for penetration scenarios under high strain rates. A specific semi-fluid transition response interval exists at the boundary between the elastoplastic penetration interval and the hydrodynamic penetration interval, where the target material exhibits both solid plastic deformation and fluid rheological characteristics. With the increase in load gradient, the stress state of the target gradually transforms from the low-stress elastoplastic stage to the high-stress fluid rheological stage, resulting in a complex and coupled global stress evolution mechanism. As the projectile velocity increases, the stress proportion corresponding to each resistance component changes dynamically. According to the pressure evolution law throughout the entire penetration process and the ultimate response characteristics of key mechanical parameters, the complete penetration behavior can be classified into three typical working conditions: rigid penetration, erosive penetration, and hydrodynamic penetration. Refined mechanical analysis of the projectile nose in different penetration stages enables the accurate calculation and fitting of the ultimate penetration depth. During rigid body penetration, the target medium is in a solid penetration state. The differential equation of the projectile’s motion can be derived [40,41,42,43]:

(1) $$\left\{\begin{array}{l} m_{p}\ddot{h}=-F\\ {\left.h\right|}_{t=0}=0\\ {\left.\dot{h}\right|}_{t=0}=v_{p0} \end{array}\right.$$

where *F* denotes the penetration resistance acting on the projectile nose. Its magnitude depends on the geometric morphology of the warhead and can be obtained by integrating the resistance components on the infinitesimal surface of the projectile nose.

**Figure 12:**
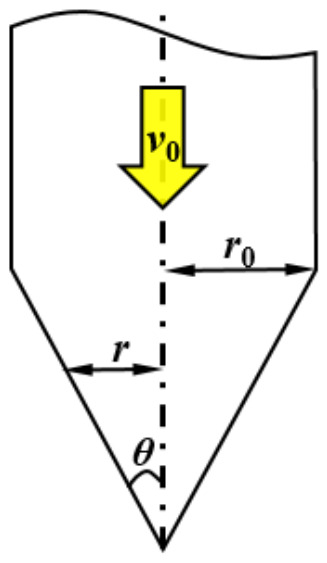
Bullet head shape.

Different nose shapes result in varying penetration resistance and penetration effects. For a conical nose, as illustrated in Figure 12, the resistance can be analyzed as follows:

A mechanical model was established for the conical projectile nose. The reference radius of the projectile was defined as *r*_0_, and the included angle between the tangent axis at any position on the outer wall of the warhead and the central axis of the projectile was denoted as *θ*. Under experimental conditions, the projectile penetrates the target vertically in a forward direction. The initial penetration velocity was defined as *v_p_*_0_, and the real-time instantaneous velocity during penetration was defined as *v_p_*. Based on the above geometric parameters and motion boundary conditions, the normal compressive resistance and tangential friction resistance acting on the infinitesimal surface of the projectile nose can be derived and solved [44,45]:

(2) $$\left\{\begin{array}{l} dF_{n}=2\pi \frac{r}{\sin\theta }\sigma_{n}d_{r}\\ dF_{t}=\mu_{s}dF_{n} \end{array}\right.$$

where *σ_n_* represents the impedance, and *µ_s_* is the friction coefficient between the projectile and the target.

The resistance *F* can be expressed as:

(3) $$F=\int_{0}^{r_{0}}dF_{n}\sin\theta +dF_{t}\cos\theta dr$$

The relationship between the velocity of particles on the target surface and the projectile velocity can be expressed as:

(4) $$v_{t}=v_{p}\sin\theta$$

Based on the above relationships, *F* can be written as:

(5) $$\left\{\begin{array}{l} F=(\alpha_{s}+\beta_{s}v_{p})\pi r_{0}^{2}\\ \alpha_{s}=\frac{4}{3}\tau sN_{p1}\\ \beta_{s}=\kappa \rho_{t}C_{t}N_{p2} \end{array}\right.$$

where *N_p_*_1_ and *N_p_*_2_ are coefficients related to the shape of the projectile. For a conical nose, these coefficients are:

(6) $$N_{p1}=\mu_{s}\cot\theta +1$$

(7) $$N_{p2}=\mu_{s}\cot\theta +\sin\theta$$

*v_p_*_0_ ≥ *v_cr_*_2_ indicates the fracture penetration stage of semi-fluid penetration. During penetration, the rod projectile fractures and penetrates simultaneously, leading to a sharp decline in kinetic energy and mass. By integrating the differential equation of projectile motion over the velocity range *v_p_*_0_~*v_cr_*_2_, the penetration depth during the fracture penetration stage is obtained as:

(8) $$X_{p1}=\frac{m_{p0}}{\rho_{t}C_{t}\pi {r^{2}}_{0}\alpha_{e2}}v_{cr2}\{1-\exp[\alpha_{e2}(1-\frac{v_{p0}}{v_{cr2}})]\}$$

*v_cr_*_2_ > *v_p_*_0_ > *v_cr_*_1_ indicates the erosion penetration stage of semi-fluid penetration. After entering the effective penetration stage, the front section of the projectile nose undergoes continuous plastic deformation and dynamic mass erosion under intense impact loading. As the penetration proceeds, the flight velocity of the projectile gradually decreases, accompanied by a continuous increase in the total erosion mass of the projectile nose material. By integrating the differential equation of projectile motion over the velocity range from *v_cr_*_2_~*v_cr_*_1_, the penetration depth during the erosion penetration stage can be determined as:

(9) $$X_{p2}=\frac{m_{p0}}{\rho_{t}C_{t}\pi {r^{2}}_{0}\alpha_{e1}}v_{cr1}\{1-\exp[\alpha_{e1}(1-\frac{v_{cr2}}{v_{cr1}})]\}$$

When the velocity decreases to *v_p_*_0_ ≤ *v_cr_*_1_, the projectile returns to rigid body penetration, and its mass no longer changes. Upon reaching the solid penetration stage with an initial velocity of *v_cr_*_1_, the penetration depth during this rigid body penetration stage can be determined as:

(10) $$X_{p3}=\frac{m_{p0}}{\pi {r^{2}}_{0}\beta_{s}}v_{cr1}\{1-\exp[\alpha_{e}(1-\frac{v_{cr2}}{v_{cr1}})]\}$$

where *m_p_*_0_ is the initial mass of the rod penetrator, *α_e_*_1_ and *α_e_*_2_ are the mass erosion coefficients, while *v_cr_*_1_ and *v_cr_*_2_ represent the critical velocities of mass erosion. The total penetration depth of the rod penetrator throughout the projectile–target interaction process is defined as:

(11) $$X_{p}=X_{p1}+X_{p2}+X_{p3}-\delta \frac{m_{p0}}{\pi {r^{2}}_{0}\beta_{s}}v_{p0}\;\mathrm{or}\;X_{p}=\frac{m_{p0}}{{d^{2}}_{0}}\delta \lambda_{1}K_{q}v_{p0}$$

In the formula, *δ* is the mass erosion coefficient.

(12) $$\delta =\frac{v_{cr}\beta_{s}-v_{cr}\beta_{s}\exp[\alpha_{e}(1-\frac{v_{p0}}{v_{cr}})]}{v_{p0}\rho_{t}C_{t}\alpha_{e}}+\frac{v_{cr}\exp[\alpha_{e}(1-\frac{v_{p0}}{v_{cr}})]}{v_{p0}}$$

**Figure 13:**
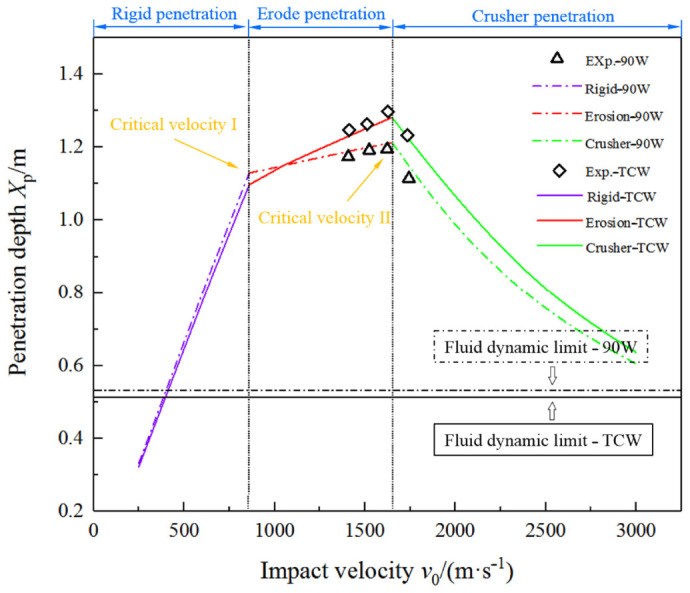
Comparison curve of penetration test data with theory and experimentation. The black solid line and black dashed line represent the hydrodynamic penetration limits of TCW and 90W, respectively.

By substituting the initial parameters of the rod projectile, the curve of the depth with velocity can be obtained, as shown in Figure 13.

During the semi-fluid penetration stage, the critical velocity for erosion penetration is 860 m/s, and the critical velocity for fracture penetration is 1650 m/s. During the erosion penetration stage, there is minimal mass loss, and the increase in velocity has a diminishing effect on penetration depth gains; during the fracture penetration stage, mass erosion dominates, causing a sharp decline in penetration depth, with the final depth approaching the hydrodynamic limit. Due to the higher sectional density of traditional tungsten alloys compared to tungsten-based ceramic alloys, traditional tungsten alloys slightly outperform in both the rigid penetration and hydrodynamic limit stages. However, when the impact velocity exceeds 1061 m/s, which occurs in the late erosion penetration stage and during the fracture penetration stage, the penetration depth of the new tungsten-based ceramic rod projectiles exceeds that of traditional tungsten alloys, and this advantage becomes more significant as the impact velocity increases. The limit penetration depth of tungsten-based ceramic alloy rod projectiles has increased by 8.85%, confirming the penetration advantages of tungsten-based ceramic alloys under ultra-high-velocity conditions. Theoretical calculations align with experimental data to within a 5% error margin.

### 4.2. Projectile Mass Erosion

**Figure 14:**
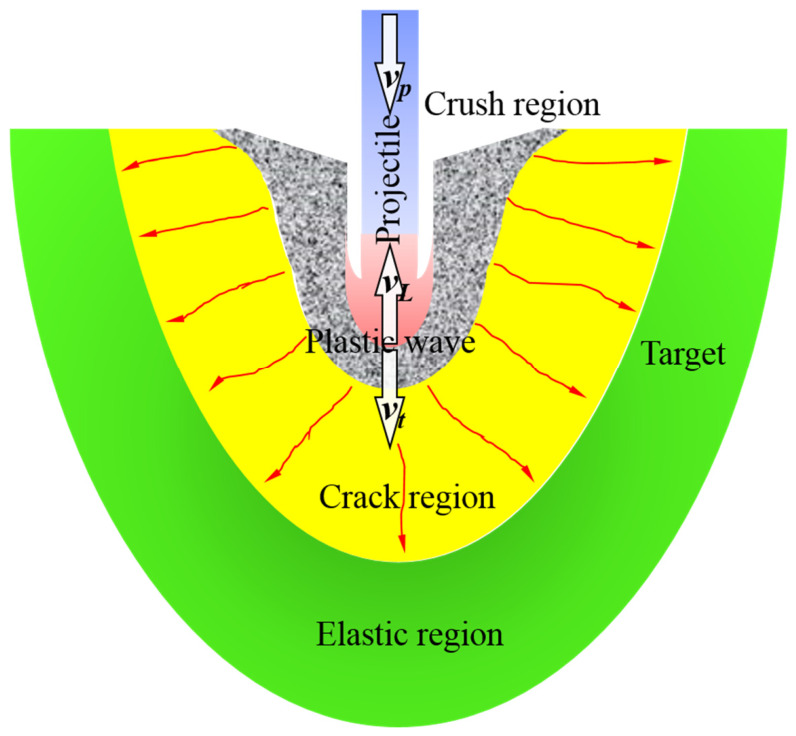
The hypervelocity intrusion projectile–target interaction process. The gray area denotes the crush region, the yellow area represents the plastic wave and crack region, and the green area is the elastic region of the target. *v_p_* is the penetration velocity of the projectile, *v_L_* represents the propagation velocity of plastic wave at the contact interface, and *v_t_* refers to the transmission velocity of stress wave inside the target. Red arrows indicate the propagation paths of plastic waves and cracks in the target material.

Through the theory combined with the test, the projectile mass change law is analyzed, and the mass erosion theoretical model is established. The theoretical model is mainly used to study the dynamics of the projectile–target interface in the process of penetration, and optimize the prediction formula for the mass loss of the projectile. According to the projectile yield model and the equation of motion, the parameters in the semi-empirical theoretical formulas are corrected using experimental data [46,47,48]. The hypervelocity intrusion projectile–target interaction process is shown in Figure 14.

When the projectile penetrates a target vertically with an initial velocity *v_p_*_0_, the densities of the projectile and target are *ρ_p_* and *ρ_t_*, and the longitudinal wave speeds are *C_p_* and *C_t_*. The contact stress caused by the penetration action is *σ_con_*. Assuming the tail velocity of the projectile during penetration is *v_p_*, the reverse motion velocity of the projectile head is *v_L_*, and the velocity of the target moving towards the penetration direction caused by the interfacial stress wave is *v_t_*, then:

(13) $$v_{p}=v_{t}+v_{L}$$

The stress at the projectile–target interface and the particle velocity is:

(14) $$\sigma_{com}=\rho_{p}C_{p}v_{L}=\rho_{t}C_{t}v_{t}$$

After organizing the above two formulas, we can obtain the following consolidated expression:

(15) $$v_{L}=\frac{v_{p}\rho_{t}C_{t}}{\rho_{t}C_{t}+\rho_{p}C_{p}}v_{t}=\frac{v_{p}\rho_{p}C_{p}}{\rho_{p}C_{p}+\rho_{t}C_{t}}$$

When *v_p_* exceeds the critical velocity, the projectile material fractures, resulting in significant mass loss. The motion equation can be formulated as follows:

(16) $$\sigma_{com}=-\frac{x\rho_{p}A_{0}}{A_{m}}\cdot \frac{dv_{p}}{dt}$$

In the formula, *A_m_* represents the cross-sectional areas of the projectile head, and *A*_0_ represents the remaining body of the projectile, while *x* is the length of the remaining projectile. *v_L_* is the velocity of erosion, defined as *v_L_* = −*dx*/*dt*. Assuming that the erosion of the projectile length is solely due to mass loss, we can eliminate *dt* to derive the following relationship:

(17) $$\frac{dx}{x}=\frac{A_{0}}{C_{p}A_{m}}dv_{p}$$

To integrate the given equation, we have:

(18) $$x=c_{1}\exp(\frac{A_{0}}{A_{m}C_{p}}V_{p})$$

By substituting the initial conditions $v_{p}=v_{p0}$ into the equation x=L and solving for the undetermined coefficient *c*_1_, we have:

(19) $$\frac{x_{r}}{L}=\exp\left[\frac{A_{0}}{A_{m}C_{p}}\left(v_{p}-v_{p0}\right)\right]$$

When the penetration *v_p_* is below the critical penetration *v_cr_*, $\alpha_{e}=A_{0}v_{cr}/A_{m}C_{p}$, the projectile enters the elastic–plastic penetration stage. At this time, the residual mass is:

(20) $$m_{r}=m_{p0}\frac{x_{r}}{L}$$

With the continuous increase in impact velocity, when the comprehensive impedance load of the target medium exceeds the dynamic yield limit of the projectile material, the projectile nose sequentially undergoes mechanical behaviors such as plastic yielding, surface material denudation, and even overall fracture failure. Under the combined effect of structural distortion and continuous mass loss of the warhead, the penetration depth presents a variation law of slightly slow increase at first and then rapid attenuation. Based on the aforementioned governing equations of penetration dynamics, a coupling correlation model between projectile mass loss and instantaneous penetration velocity was introduced [49], which can further derive the real-time instantaneous mass expression of the projectile during the entire penetration process.

(21) $$m_{r}=\left\{\begin{array}{l} m_{p0}\;\;(v_{0}\leq v_{cr1})\\ m_{p0}\exp[\alpha_{e1}(1-\frac{v_{p0}}{v_{cr1}})]\;\;(v_{cr1}<v_{0}<v_{cr2})\\ m_{p0}\exp[\alpha_{e2}(1-\frac{v_{p0}}{v_{cr2}})]\;\exp[\alpha_{e1}(1-\frac{v_{cr2}}{v_{cr1}})]\;\;(v_{0}\geq v_{cr2}) \end{array}\right.$$

**Figure 15:**
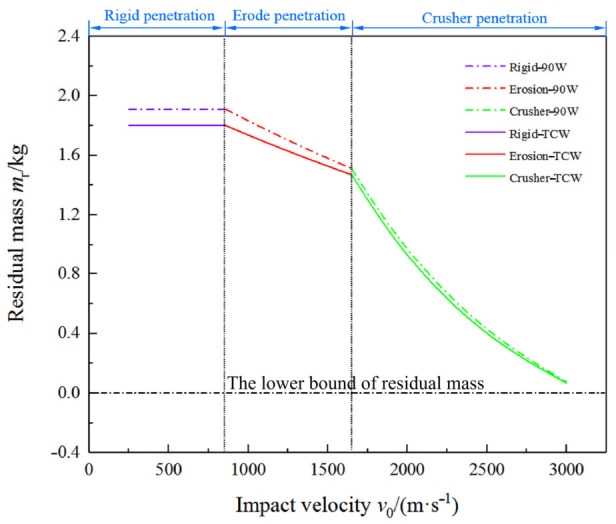
Mass versus velocity curve of the projectile. The black dashed line represents the condition of complete mass erosion and loss, where the residual projectile mass equals zero, serving as the lower bound of residual mass.

Based on the curves depicting the changes in the residual mass of rod projectiles made from the two materials as a function of velocity, as shown in Figure 15, the critical transition velocity is essentially consistent. During the erosive penetration phase, where the primary erosion occurs at the projectile’s tip, transitioning from a conical to a hemispherical shape results in a reduction in mass. The average mass erosion rate at this stage is 0.51 kgs/km. As the initial velocity increases further, the penetration mechanism changes, and the mass loss primarily results from significant deformation and fracture at the front end, with an average mass erosion rate of 1.06 kgs/km.

Comparing the residual mass of the projectiles made from the two materials after penetration reveals that during the erosive penetration phase, the average mass loss rate for the tungsten-based ceramic rod projectiles is 18.33%, which is 12.09% lower than that of the tungsten alloy rod projectiles. The average erosion rate for the tungsten-based ceramic rod projectiles is 0.42 kgs/km, which is 17.65% lower than that of the tungsten alloy rod projectiles. The introduction of sintered ceramic particles effectively reduces mass erosion during the ultra-high-speed penetration process. However, this advantage becomes less significant in the fracture penetration phase, where the residual mass of projectiles made from both materials essentially reduces to 0 at the fluid dynamics limit.

## 5. Conclusions

Aiming at the problem of penetration depth attenuation caused by the lateral deformation effect of tungsten alloys during ultra-high-velocity penetration, a lightweight tungsten-based ceramic alloy is fabricated in this study. The ultra-high-velocity penetration performance of the novel material is systematically verified via ballistics tests, and the evolution laws of projectile penetration depth and residual mass at different penetration velocities are revealed through theoretical prediction. The main conclusions are summarized as follows:

(1) Lightweight tungsten-based ceramic alloy composites were fabricated and subjected to mechanical property tests to characterize the mechanical behaviors of the new material. Compared with the monolithic 90W reference alloy, the tensile strength of the tungsten-based ceramic alloy is increased by 13.64%, while its ductility is reduced by 43.75%. Microstructural and fracture morphology analyses reveal that transgranular cleavage dominates the failure mode of the composite. During crack propagation, ceramic microspheres hinder crack growth, forcing new cracks to nucleate or altering the propagation path of existing cracks.
(2) Based on the existing theoretical framework, the semi-fluid penetration stage is subdivided into the erosion-dominated penetration stage and fragmentation-dominated penetration stage. A three-stage theoretical model for ultra-high-velocity penetration is proposed accordingly. The penetration depth curves and residual projectile mass curves of rod projectiles made of the two materials penetrating concrete targets at different penetration stages are derived. The error between the theoretical model predictions and experimental data is within 5%.
(3) Ballistics test results fully confirm that the lightweight tungsten-based ceramic alloy exhibits excellent performance advantages in the semi-fluid penetration stage. With the increase in penetration velocity, the penetration gain of the composite is continuously improved. Compared with conventional tungsten alloys, its dimensionless penetration depth is increased by 10.58%, while the average projectile mass loss rate and average erosion rate are reduced by 12.09% and 17.65%, respectively. Benefiting from its superior ultra-high-velocity penetration capability, wear resistance and impact resistance, the lightweight tungsten-based ceramic alloy can be widely applied in various engineering fields, including civil emergency obstacle clearance, ultra-deep drilling in geotechnical engineering, rapid demolition of waste buildings, and deep geological sampling, presenting promising civilian application prospects.

## Figures and Tables

**Table 1:** Mechanical properties of tungsten-based ceramic alloys.

State	Density /(g/cm^3^)	Tensile Strength R_m_/(MPa)	Elongation A/(%)	Reduction in Area Z/(%)	Impact Toughness A_KU_/(J)
90W-Ni-Fe	17.06	972.2	16.1	12.1	52.4
	17.12	985.5	16.8	12.5	55.8
	17.08	991.7	17.0	12.7	56.9
	17.14	978.1	16.3	12.2	53.2
	17.10	974.5	16.8	12.5	54.7
90W-Ni-Fe-2.5%Al_2_O_3_	15.76	918.3	5.2	6.8	19.4
	15.82	931.8	5.4	7.0	20.5
	15.79	929.1	5.3	6.9	21.8
	15.83	922.6	5.1	6.7	19.2
	15.80	930.2	5.5	7.1	21.1

**Table 2:** *C* values at different strain rates.

Material	Parameter	*C*_1_	*C*_2_	*C*_3_	*C*_4_	*C*_5_	*C*
90W-Ni-Fe	Strain rate/s^−1^	2400	3800	4800	5900	7600	/
	Coefficient *C*	0.0198	0.0365	0.0447	0.0387	0.0325	0.0342
90W-Ni-Fe-2.5%Al_2_O_3_	Strain rate/s^−1^	2500	3700	5100	6200	7600	/
	Coefficient *C*	0.0214	0.0397	0.0511	0.0517	0.0672	0.0462

**Table 3:** Test records of rod ammunition penetrating concrete targets.

Group	Material	Impact Velocity *v*_0_/(m/s)	Projectile Mass *m*_1_/ (kg)	Propellant Mass *m*_2_/(kg)	Penetration Depth *X*_p_/(m)	Penetration Increase /(%)	Subsequent Target	
							Diameter/(mm)	Depth/(mm)
W-I	90W	1408.8	1.911	6.75	1.174	6.22	48 × 98	174
T-I	TCW	1414.1	1.801	6.65	1.247		31 × 49	247
W-II	90W	1524.3	1.912	7.50	1.182	6.94	70 × 73	182
T-II	TCW	1511.5	1.803	7.20	1.264		67 × 55	264
W-III	90W	1623.8	1.910	7.85	1.195	8.62	99 × 84	195
T-III	TCW	1626.9	1.801	7.43	1.298		81 × 71	298
W-IV	90W	1743.5	1.912	8.55	1.115	10.58	130 × 230	115
T-IV	TCW	1735.6	1.802	8.30	1.233		Ø100	233

## Data Availability

The original contributions presented in this study are included in the article. Further inquiries can be directed to the corresponding authors.

## Dual-Use Research Statement

The current research is confined to the field of ultra-high-speed penetration dynamics of lightweight tungsten-based ceramic alloy projectiles. This study focuses on the dynamic response mechanism and penetration efficiency law of projectile–target interaction, which belongs to basic applied research in the field of impact engineering and protective engineering. The research has essential practical value for the optimal design of protective concrete structures and the performance improvement of lightweight penetration components, and poses no potential threat to public health, social safety, and national security. The authors fully acknowledge the dual-use research attributes of ultra-high-speed impact and penetration technology. All experimental schemes, numerical simulation methods, and data analysis processes adopted in this study are limited to academic research and engineering application verification in the field of protection engineering. No dangerous technology routes, sensitive processing parameters, or executable weapon manufacturing core technologies are involved in the manuscript. All authors strictly abide by relevant national laws, regulations and international academic norms governing Dual Use Research of Concern (DURC) and have implemented comprehensive preventive measures to avoid inappropriate use and malicious misuse of the research results. The academic and engineering benefits of this study far outweigh potential adverse risks. On the one hand, this research systematically reveals the ultra-high-speed penetration failure mechanism of lightweight tungsten-based ceramic alloy projectiles against concrete targets, fills the research gap in the dynamic performance evaluation of new composite penetration materials, and provides a theoretical basis and technical reference for the lightweight and high-efficiency optimization of impact-resistant components. On the other hand, the research conclusions can guide the anti-penetration structural optimization of civil and national defense concrete protection projects, improve the safety and protection performance of engineering structures, and produce positive social and engineering value. All research data and conclusions are publicly reported transparently for academic exchange and engineering research purposes only, effectively standardizing the application of the research results and eliminating potential misuse risks.
